# Ultra-High Resolution Inelastic Neutron Scattering

**DOI:** 10.6028/jres.098.007

**Published:** 1993

**Authors:** D. A. Neumann, B. Hammouda

**Affiliations:** National Institute of Standards and Technology, Gaithersburg, MD 20899

**Keywords:** diffusion, inelastic neutron scattering, molecular reorientations, neutron backscattering spectrometers, neutron spin-echo spectrometers, polymer dynamics, rotational tunneling

## Abstract

Two types of ultra high energy resolution neutron scattering instruments, the backscattering spectrometer and the spin echo spectrometer, are described. Examples of the types of research which can be done with these instruments are given and plans for a cold neutron backscattering spectrometer which will be built in the NIST Cold Neutron Research Facility (CNRF) are discussed. It is hoped that this information will be of use to researchers considering neutron scattering experiments at NIST.

## 1. Introduction

Neutron scattering has proven to be an extremely valuable tool for studying a wide variety of dynamical processes in solids. This is because the energy of thermal neutrons is comparable to the energies of many elementary excitations in condensed matter while their wavelength is comparable to the interatomic distances. This allows one to simultaneously obtain information on the time scale and the geometry of the dynamical process under study; a feature which is unmatched by any other technique [1–6]. An important time scale for the investigation of dynamical phenomena such as diffusion, molecular reorientations and molecular tunneling is 10^−7^ to 10^−9^ s, the regime of ultrahigh resolution inelastic neutron scattering. The first neutron scattering instrument to operate in this range was a backscattering spectrometer built at the Munich reactor around 1970 which had an energy resolution of 0.425 µeV [7]. In the next section we describe how this excellent energy resolution is obtained and give a schematic description of a backscattering spectrometer. We also describe the spin-echo spectrometer which was first proposed in 1972 [8]. The following section describes the basic theory and gives examples of the use of quasielastic scattering to determine both the time scale and spatial character of diffusion and molecular reorientations. We then go on to discuss rotational tunneling and show how neutron scattering measurements can yield detailed information on the orientational potential felt by molecules in condensed systems. The final section describes the conceptual design of the backscattering spectrometer to be built in the CNRF at NIST and the basic design goals for a spin-echo spectrometer.

## 2. Fundamentals

### 2.1 Types of Instruments

#### 2.1.1 Backscattering

**Fig. 1:**
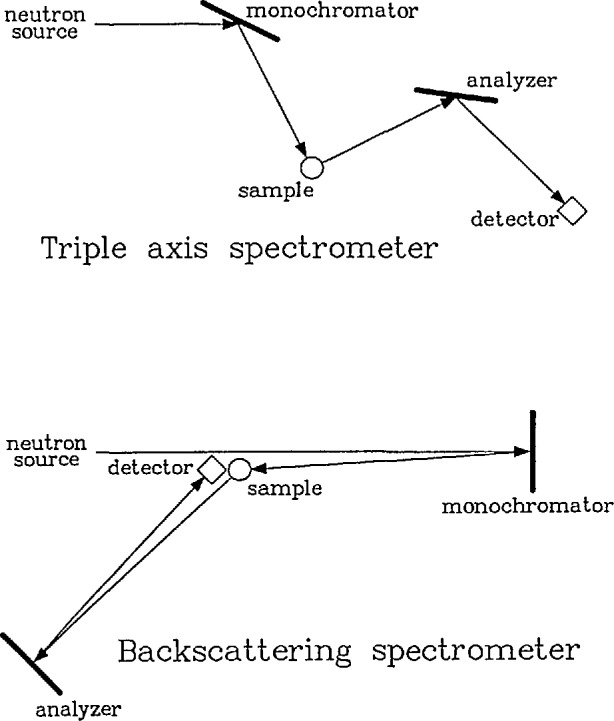
Schematic diagrams showing the relationship between a triple-axis spectrometer and a backscattering spectrometer. Note that the term “backscattering” refers to the scattering from the monochromator and analyzer crystals and not from the sample.

A neutron backscattering spectrometer is closely related to the triple axis spectrometer [9] shown schematically in Fig. 1. In both types of instruments, a “white” beam of neutrons impinges on a monochromator crystal which selects a given neutron wavelength by Bragg diffraction. The resulting monochromatic neutrons then scatter from the sample, possibly gaining or losing energy in the process. The energies of the scattered neutrons are determined by Bragg diffraction from an analyzer crystal. The excellent energy resolution is obtained by taking the triple-axis instrument to its extreme limit, i.e., scattering the neutrons through an angle of 180° at both the monochromator and analyzer crystals. The energy resolution δ*E* for a single Bragg reflection can be found by differentiating Bragg’s law. One then obtains

$$\frac{\delta E}{E}=\frac{2\delta d}{d}+2\cot\;\theta \;\delta \theta ,$$ (1)

where δ*d* and *d* are the spread and value of the *d* spacing of the particular lattice planes used for monochromating or analyzing the neutron energy *E, θ* is 1/2 the scattering angle, and δ*θ* is the angular spread of the neutrons. For a backscattering instrument the second term is zero since *θ* is 90°. Thus the resolution is determined by δ*d/d*, which for perfect crystals is given by the Darwin width which is roughly on the order of 10^−5^. Of course the beam is not perfectly collimated so that the energy resolution in backscattering is actually given by [7]

$$\frac{\delta E}{E}=2\frac{\delta d}{d}+\frac{1}{4}{\left(\delta \theta \right)}^{2}.$$ (2)

Thus, using the backscattering geometry for both the monochromator and analyzer, can result in energy resolutions ≲0.1 μeV.

There are obvious technical difficulties inherent in the backscattering geometry. For instance one cannot simply scan energy by changing the scattering angle of the monochromator or analyzer since to do so would result in moving away from the backscattering condition. This is overcome by changing the incident energy, *E_i_*, by either Doppler shifting the incident neutrons by rapidly moving the monochromator crystals or by changing the *d*-spacing of the monochromator crystal as a function of time using thermal expansion. There are several other difficulties including the low intensity and the geometry of the sample detector-area which will be discussed later in this article where the plans for the NIST cold-neutron backscattering instrument are described in detail.

#### 2.1.2 The Neutron Spin Echo

**Fig. 2:**
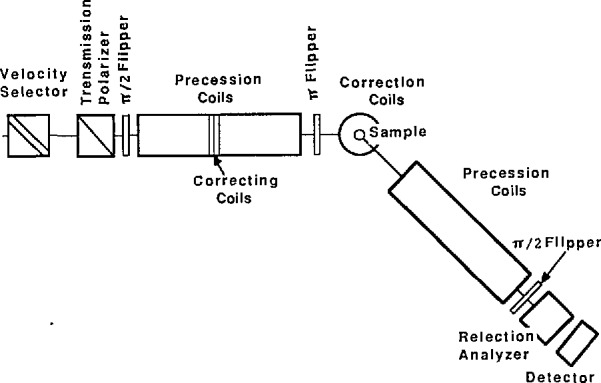
Schematic diagram of a spin-echo spectrometer.

The NSE technique [8] uses the Larmor precession of the neutron spin to measure the change in the energy of the neutron upon scattering from some dynamical process in condensed matter. The idea is to make polarized neutrons precess in “very” uniform opposite magnetic fields before and after the sample so that those having slightly different wavelengths end up with the same spin orientation at the analyzer position. This allows the realization of excellent energy resolutions (≲1 μeV) using typical (i.e., broad) neutron wavelength distributions. As indicated in Fig. 2, cold neutrons are first polarized, then made to precess in very uniform magnetic fields in one direction before the sample and in the other direction after the sample and finally their spin orientations are analyzed to obtain the angular shift introduced by the sample on the spin orientation. This angular shift a is proportional to the applied magnetic field *H*, to the precession length *L* and to the energy shift during scattering *ℏω* = *E*_f_− *E*_i_:

$$\alpha =\left[\gamma h^{2}{\left(\pi /m\right)}^{1/2}\right]\hbar \omega \lambda^{3}HL,$$ (3)

where *γ,m,h* and λ are the gyromagnetic constant, the neutron mass, Planck’s constant, and the neutron wavelength, respectively. The analyzer picks up the projection of the magnetic moment along a well defined direction so that the detected neutron intensity is proportional to cos(α) properly weighted over the normal modes distribution *S*(*Q, ω*). The measured intensity is therefore given by the cosine Fourier transform of the dynamic structure factor *S*(*Q, ω*) [6]:

I(Q,t)=∫dωS(Q,ω)cos(α), (4)

where the Fourier variable is *t* =[γ*h*^3^/2(πm)^1/2^] *λ*^3^*HL.* In order to scan time, the magnetic field *H* is varied.

The main components of an NSE instrument [10,11] (see Fig. 2) are the supermirrors to polarize and analyze the neutron spin direction, the coils to create very uniform magnetic fields (δ*H*/*H* ~ 10^−5^) and other conventional neutron scattering components (velocity selector to monochromate, slits to collimate and a detector to count scattered neutrons). Flippers are used to prepare the neutron spin direction before, and after the two precession coils by rotating its direction.

### 2.2 Quasielastic Neutron Scattering

#### 2.2.1 Basic Theory

In this section we first outline basic features of quasielastic neutron scattering and then proceed to illustrate these points with various experimental applications. To date, most quasielastic neutron scattering experiments have been performed using incoherent scattering [6], due to the simpler interpretation in terms of specific microscopic models. This is because the incoherent scattering function *S*_inc_(***Q***, *ω*) measured by the backscattering spectrometer is the space and time Fourier transform of the self-correlation function *G_s_*(***r***,*t*) which represents the probability that a particle which was at the origin at time *t* =0 is at position ***r*** at time *t.* Thus when devising a model of a diffusional process, it is only necessary to con-sider the motion of a single atom and not how the motion of that atom is correlated with the motions of other atoms in the system. Another important way of expressing *S*_inc_(***Q***, *ω*) is in terms of the intermediate scattering function *I*(***Q***,*t*)

$$S_{\mathrm{inc}}\left(Q,\;\omega \right)=\int I\left(Q,t\right)e^{i\omega t}dt,$$ (5)

where *I*(***Q***,*t*) is the space Fourier transform of *G_s_*(***r***,*t*) and is the quantity measured by the spin-echo technique.

In order to understand qualitatively how diffusion is manifested in a neutron scattering experiment, we will consider some simple models which display all of the basic features of more complex models. (For a more detailed discussion of the models presented here and for a far wider assortment of models see [5]). First consider simple diffusion which is governed by Fick’s law

$$\frac{\partial \rho \left(r,t\right)}{\partial t}=D\nabla^{2}\rho \left(r,t\right),$$ (6)

where *p*(***r***,*t*) is the particle density at position ***r*** at time *t* and *D* is the diffusion constant. A solution of this equation is given by a self-correlation function of the form

$$G_{s}\left(r,t\right)=\frac{\exp\left(-r^{2}/4Dt\right)}{{\left(4\pi Dt\right)}^{3/2}},$$ (7)

where we have assumed that the times of interest are long enough that the motion is truly diffusive, i.e., much longer than the time between collisions. Then the space Fourier transform of Eq. (6) yields the intermediate scattering function

$$I\left(Q,\;t\right)=\exp\left(-Q^{2}Dt\right)$$ (8)

shown in Fig. 3a.

**Fig. 3:**
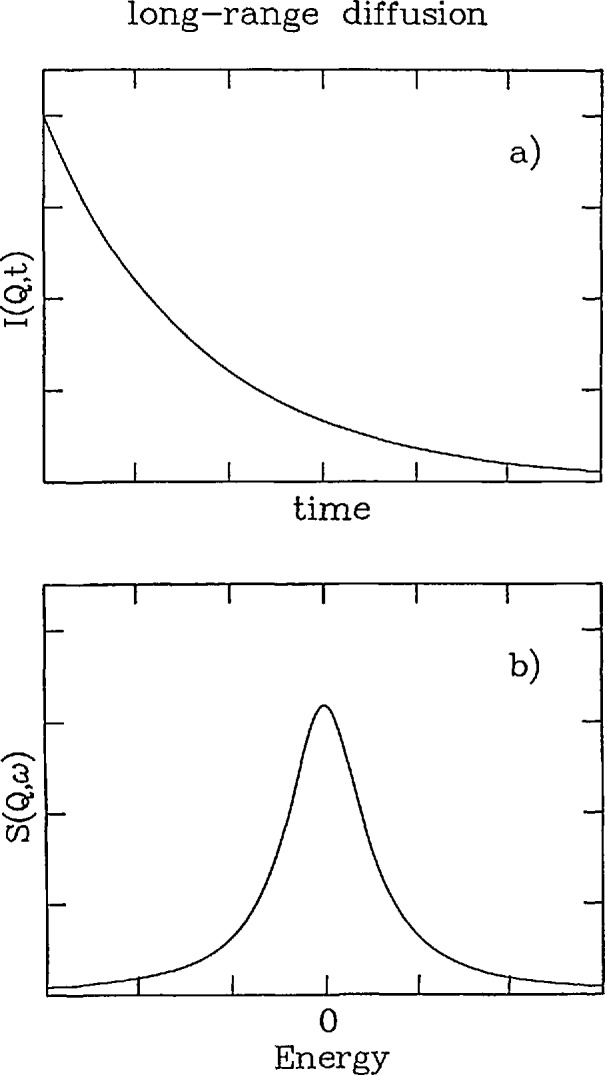
a) Intermediate scattering function of time at a particular value of the scattering vector ***Q***.b) Scattering function for long-range translational diffusion as a function of energy at a particular *Q.* This is the Fourier transform of the intermediate scattering function.

Since this represents an exponential decay in time, the time Fourier transform yields a Lorentzian lineshape

$$S_{\mathrm{inc}}\left(Q,\omega \right)=\frac{1}{\pi }\frac{DQ^{2}}{{\left(DQ^{2}\right)}^{2}+\omega^{2}},$$ (9)

which is shown in Fig. 3b. Note that this expression peaks at *ω* = 0 and has an energy width (FWHM) *T* which is given by

$$\Gamma =2DQ^{2}.$$ (10)

**Fig. 4:**
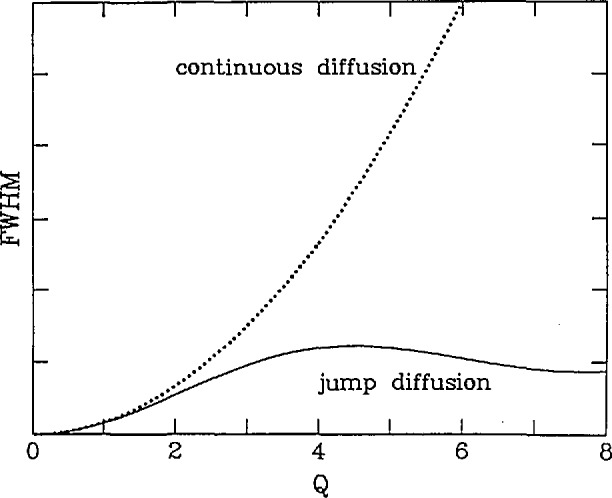
Full width at half maximum (FWHM) for Fickian (continuous) diffusion (dotted line) and for the Chudley-Elliott model of translational jump diffusion for 1 Å jumps (solid line). Note that they are identical at low *Q* which means that the macroscopic diffusion constants are identical.

The width of the peak is thus proportional to both the diffusion constant and the square of the scattering vector as shown in Fig. 4.

Chudley and Elliott [12] generalized this picture to describe jump diffusion in solids by assuming that the jump motion is random, that the jumps can be considered instantaneous, and that the available lattice sites form a Bravais lattice. Then the simple rate equation

$$\frac{\partial \rho \left(r,t\right)}{\partial t}=\frac{1}{n\tau }\sum_{i=1}^{n}\;\left[\rho \left(r+\ell_{i},t\right)-\rho \left(r,t\right)\right],$$ (11)

can be used to represent the particle’s motion. Here *ρ*(***r***,*t*) is the probability of finding the particle at position ***r*** at time, *t*, *τ* is the time between jumps and the sum is taken over the nearest neighbor sites at distances *ℓ_i_*. Using the boundary condition *ρ*(***r***, 0) = δ(***r***) makes *ρ*(***r***,*t*) and *G_s_*(***r***,*t*) equivalent, and then the Fourier transform of the previous equation yields

$$\frac{\partial I\left(Q,t\right)}{\partial t}=\frac{-I\left(Q,t\right)}{\tau }\frac{1}{n}\sum_{i=1}^{n}\left(1-e^{-iQ\cdot n}\right).$$ (12)

As for the case of pure diffusive motion, this has an exponential solution of the form

$$I_{s}\left(Q,t\right)=\exp\left(-\frac{f\left(Q\right)t}{\tau }\right),$$ (13)

where

$$f\left(Q\right)=\frac{4}{n}\sum_{i=1}^{n/2}\sin^{2}\;\left(\frac{Q\cdot \ell_{i}}{2}\right).$$ (14)

Thus the scattering function again has a Lorentzian lineshape given by

$$S_{\mathrm{inc}}\left(Q,\omega \right)=\frac{1}{\pi }\frac{f\left(Q\right)/\tau }{{\left(f\left(Q\right)/\tau \right)}^{2}+\omega^{2}},$$ (15)

which has an energy width of

$$\Gamma =\frac{2}{\tau }f\left(Q\right).$$ (16)

The most important thing to note is that *Γ* oscillates in ***Q*** with the periodicity determined by the inverse of the jump vectors *ℓ_i_*. Thus it is possible to determine the microscopic diffusion mechanism via the dependence of the width of the quasielastic scattering on the scattering vector. Another interesting feature is that for small values of ***Q***, *Γ* ∝ *Q*^2^/τ. One can then connect this expression to the macroscopic diffusion constant since *D* ∝ 1/*τ* and *Γ* = 2*DQ*^2^ for Fickian diffusion. Fig. 4 compares *Γ*(***Q***) for a powder averaged Chudley-Elliott model assuming l Å jumps with that of Fickian diffusion for identical values of the diffusion constant. The possibility of extracting the macroscopic diffusion constant from the small *Q* region makes it possible to compare quasielastic neutron scattering results with those obtained using other methods and to discern the activation energy *E_0_* via the Arrhenius law

$$D=D_{0}\;\exp\left(\frac{-E_{0}}{k_{B}T}\right).$$ (17)

**Fig. 5:**
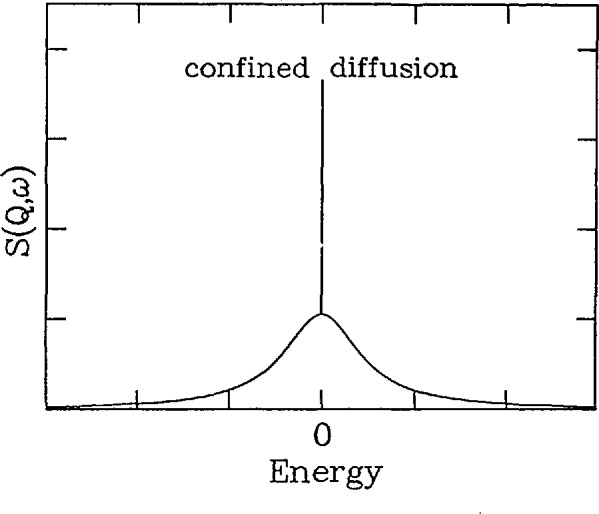
Scattering function as a function of energy at a particular *Q* for diffusion confined to a particular region of space (e.g., rotational jump diffusion). Note the narrow component indicative of a process in which the atom has a finite probability of being at its initial position at infinite time.

For rotational motions, one is typically concerned with molecules or ions which contain more than one hydrogen atom. Thus it should be reiterated that to describe the motion, only a single atom need be considered for an incoherent scatterer, since the motions of other atoms are irrelevant even if they are coupled to that of the first [6]. The formalism for rotational motions is thus the same as for diffusion in which a single particle is confined to a limited region of space. First let us turn our attention to the case in which an atom undergoes jump diffusion on a limited number of sites which lie on a circle of diameter *R.* Consider functions *f_i_*(***t***) which represent the probability that a particular atom is at site *i* at time *t.* These functions may be obtained using a rate equation similar to Eq. (10)

$$\frac{df_{i}\left(t\right)}{dt}=\frac{1}{\tau }f_{i}\left(t\right)+\frac{1}{\tau }\sum_{j\neq 1}f_{j}\left(t\right),$$ (18)

where τ is the time between jumps and the sum is taken over all orientations from which the molecule can rotate directly to orientation *i.* For simplicity we will consider the case of two possible equivalent molecular orientations corresponding, for example, to a water molecule undergoing twofold jumps about its *C*_2_ symmetry axis. Then Eq. (17) has the solutions

$$f_{1}=\frac{1}{2}\left(1+\exp\frac{-2t}{\tau }\right)$$ (19)

$$f_{2}=\frac{1}{2}\left(1-\exp\frac{-2t}{\tau }\right),$$ (20)

where use has been made of the relations *f*_1_(0) = 1, *f*_2_(0) = 0 and *f*_1_+*f*_2_ = 1. The intermediate scattering function is then given by

$$\begin{array}{c} I\left(Q,t\right)=\frac{1}{2}\exp\;\left(\frac{-2t}{\tau }\right)\times \\ \left[1-\exp\;\left(iQ\cdot R\right)\right]+\frac{1}{2}\left[1+\exp\;\left(iQ\cdot R\right)\right], \end{array}$$ (21)

where ***R*** is the vector between positions 0 and 1. Note that this equation has been divided into two parts. The first decays exponentially in time and thus leads to a Lorentzian component in the quasielastic scattering while the second is independent of time and, therefore, gives a δ-function in energy. This lineshape is displayed in Fig. 5. After performing a three-dimensional powder average and a Fourier transform, one obtains the scattering function

$$\begin{array}{l} S_{\mathrm{inc}}\left(Q,\omega \right)=\frac{1}{\pi }[\frac{1}{2}\left(1+\frac{\sin\left(QR\right)}{QR}\right)\delta \left(\omega \right)+\\ \;\left(1-\frac{\sin\left(QR\right)}{QR}\right)\frac{2\tau }{{\left(2\right)}^{2}+{\left(2\omega \tau \right)}^{2}}\;]. \end{array}$$ (22)

Note that for rotational jump diffusion, the Lorentzian component has a linewidth which is constant in ***Q***, but that the intensity oscillates with the inverse periodicity of the jump length. The intensity of the δ-function component, termed the elastic incoherent structure factor or EISF, oscillates with the same period but out of phase with respect to the intensity of the Lorentzian component. Thus, characteristic differences exist in the scattering from rotational jump diffusion compared to translational jump diffusion where the lineshape is a single Lorentzian. It is worth pointing out that the δ-function component arises from the fact that at infinite time, the particle has a finite probability of being in its original position. Thus this δ-function component is a characteristic feature of any diffusion process which is confined to a specific region of space.

**Fig. 6:**
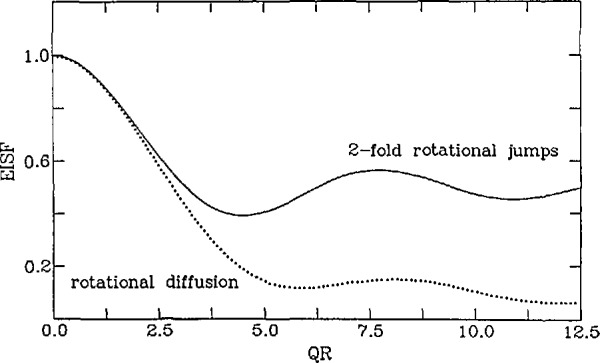
The elastic incoherent structure factor (EISF) for uniaxial twofold rotational jumps (solid line) and for uniaxial rotational diffusion (dotted line) as a function of ***QR*** where ***R*** is the diameter of the circle on which the motion occurs.

The microscopic rotational mechanism need not be as well-characterized as it was for this simple example. For instance, if the static potential fluctuates due to phonons, the idea of a single jump frequency needs to be replaced by a distribution of residence times. This situation is called rotational diffusion since the self-correlation function obeys the diffusion equation if the residence time is short. Then for uniaxial rotational diffusion, it can be shown that

$$\begin{array}{l} S_{\mathrm{inc}}\;\left(Q,\omega \right)=J_{0}^{2}\left(\frac{QR}{2}\;\sin\theta \right)\delta \left(\omega \right)+\\ \;\frac{2}{\pi }\sum_{i=1}^{\infty }J_{i}^{2}\left(\frac{QR}{2}\;\sin\theta \right)\;\frac{\Gamma_{i}}{\Gamma_{i}^{2}+\omega^{2}}, \end{array}$$ (23)

where *R* is the diameter of circle on which the diffusion is occurring, *θ* is the angle between the axis of rotation and *Q* and *Γ_i_ =i^2^D_R_* with *D*_R_representing the rotational diffusion constant. Thus, the scattering function can still be divided into a completely elastic component and a broadened component. However, in this case, the broadened component is a sum of many different Lorentzians of varying widths. Therefore, the total width of this component may vary somewhat in *Q* due to the trade-off in intensity between the various Lorentzians. The EISF’s of the two models discussed here are displayed in Fig. 6. Note that for the case of twofold jumps, the EISF decays to 1/2 at large values of *Q*. This is simply a manifestation of the fact that the EISF represents the Fourier transform of the self-correlation function for infinite times. For a two-site model the probability is 0.5 that the particle has its original orientation, therefore the EISF only drops to 1/2, but for the rotational diffusion model the EISF eventually drops to zero since there are infinitely many possible sites on a circle. In principle it is possible to tell if a particle is undergoing rotational jumps or continuous rotational diffusion on this basis alone. In practice one usually cannot reach *Q*’s which are high enough to distinguish continuous diffusion from discrete many-fold jumps.

**Fig. 7:**
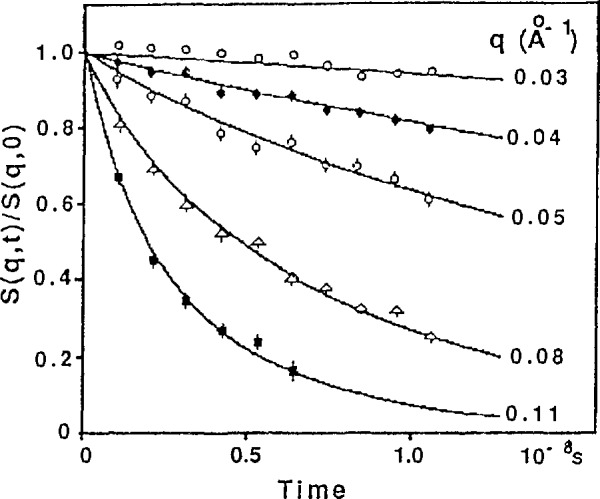
Neutron spin eeho spectra from polydimethylsiloxane in a dilute solution of deuterated bromobenzene at the theta temperature (84 °C) [13].

In polymer research the intermediate scattering function *I*(*Q,t*) is often expressed as the density-density correlation between monomers:

$$\begin{array}{c} I\left(Q,t\right)=\left(1/N_{P}N^{2}\right)\sum_{\alpha \beta }^{N_{P}}\sum_{ij}^{N}\\ <\exp\left[-iQ\cdot \left(r_{\alpha i}\left(0\right)-r_{\beta j}\left(t\right)\right)\right]>, \end{array}$$ (24)

where *r_βj_*(*t*) is the position of monomer *j* in polymer *β* at time *t*, *N_p_* and *N* are the total number of polymers and the number of monomers per polymer chain respectively. Its initial value is the elastic (also called static) structure factor: *S*(*Q*,0)*=I*(*Q*,t = 0). Fig. 7 shows a typical set of neutron spin echo (NSE) data taken by Ewen [13] from polydimethyl-siloxane (PMDS) in a dilute solution of deuterated bromobenzene at the Theta temperature (84 °C). Note that these curves exhibit the simple exponential behaviour displayed schematically in Fig. 3a. Furthermore, Eq. (8) shows that the decay of *I*(*Q*,*t*) increases with *Q*^2^ which is also observed in Fig. 7.

The initial slope of *I*(*Q*,*t*) is called the first cumulant:

$$\Gamma \left(Q\right)=\underset{t\to 0}{\mathrm{Lim}}\left[\partial I\left(Q,t\right)/\partial t\right]/S\left(Q,0\right),$$ (25)

which is identical to the energy width of *S*(*Q*,*ω*) and which can readily be modelled for various polymer systems. Many approaches are used: the Kirkwood-Riseman (KR) equation for polymer solutions, the dynamic Random Phase Approximation [14] (RPA) for polymer melts, scaling concepts and renormalization group ideas for both, etc.

The precision of extracting *Γ*(*Q*) is greatly improved by introducing a shape function [15, 16]

f(Q,τ)=I(Q,t)/S(Q,0), (26)

where time is rescaled by defining a dimensionless variable *τ = Γ*(*Q*)*t*. This function depends only on *QR_g_* or *Qa* in the small or high *Q* regions (where *R_g_* is the radius of gyration and *a* is the statistical segment length). Moreover, it is independent of *Q* in the intermediate *Q* region which means that the scattering function follows a universal shape (the intermediate *Q* region is defined as *1/R_g_* < *Q < 1/a).* An iterative procedure using the shape function *f*(*Q,τ*) yields values for *Γ*(*Q*) that are more precise than the direct method based on simply extracting *Γ*(*Q*) as the slope of *I*(*Q,t*) at zero time.

**Fig. 8:**
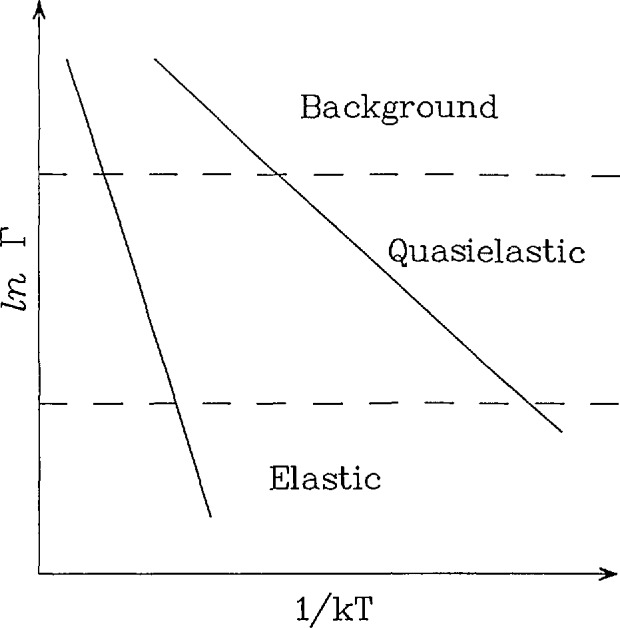
Schematic Arrhcnius plots showing that a motion occurring on a particular time scale can give rise to scattering which appears elastic if the instrumental resolution is too coarsc, while it may appear as a flat background if the resolution is too fine. This indicates that motions which occur on different time scales can be separated simply by using instruments having different dynamical ranges or by changing the resolution on a given instrument.

Many times a system will display more than one type of diffusive motion; then if the various motions are uncoupled, the intermediate scattering function is given by the product of the individual intermediate scattering functions

$$I\left(Q,t\right)=I^{\mathrm{vib}}\left(Q,t\right)\;\underset{j}{\Pi }\;I_{j}\left(Q,t\right),$$ (27)

where the product is taken over the various rotational and translational motions and

$$I^{\mathrm{vib}}\left(Q,t\right)=\exp\left(-Q^{2}<\mu^{2}>\right)$$ (28)

is simply the Debye-Waller factor. This results in a scattering function which is simply the convolution of the scattering functions of the individual motions. Thus if the motions occur on somewhat different time scales, the various components can often be separated simply because they have different widths (Fig. 8). This is possible because motions which are slow on the scale of the resolution will appear as an elastic component and those which are fast compared to the resolution will appear as an essentially flat background. In order to observe motions occurring on different time scales usually means using different instruments with different dynamical windows or at least adjusting the resolution on a given instrument. Thus it is often important to have a wide dynamical range available in order to completely characterize a diffusional process.

#### 2.2.2 Applications

##### Li Diffusion in LiC_6_

**Fig. 9:**
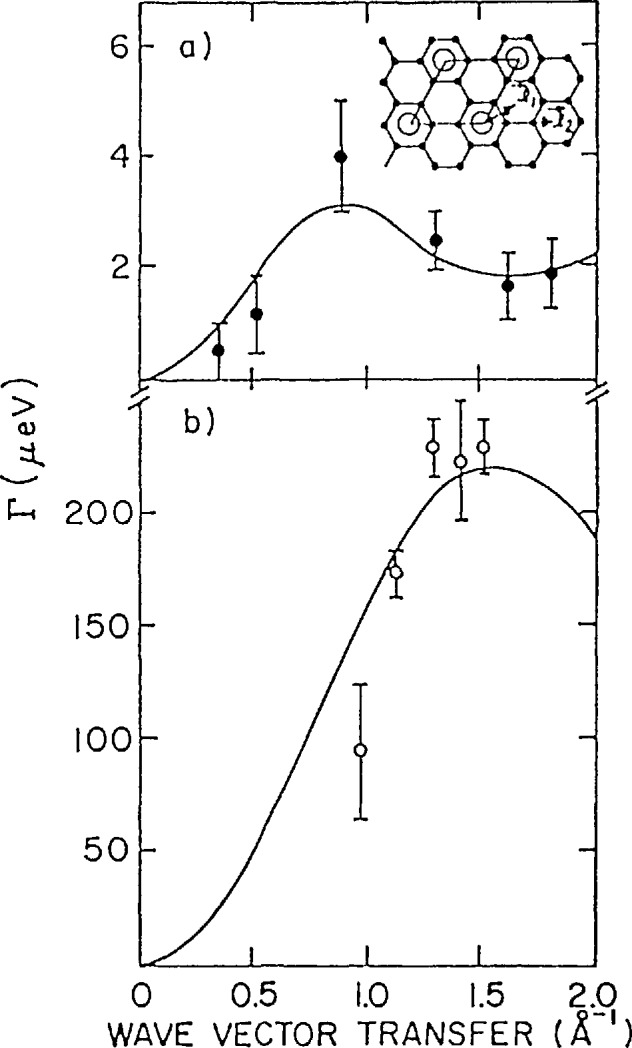
Linewidths (FWHM) of the quasielastie neutron spectra measured for LiC_6_. a) 660 K (below the Li sublattiee melting temperature), b) 720 K (above the Li sublattiee melting temperature). The inset shows the jump vectors used to calculate the solid lines in both plots. Above the transition, Li jumps to nearest neighbor sites while below jumps oceur to sites form the $\sqrt{3}\times \sqrt{3}$ R30° sublattice [18].

LiC_6_ is a stage 1 graphite intercalation compound in which the Li atoms undergo a transition from an ordered, commensurate $\sqrt{3}\times \sqrt{3}$ R30° phase to a disordered, commensurate lattice gas at a temperature of 715 K [17]. Measurements of the quasielas-tic scattering due to Li diffusion were made by Magerl, Zabel, and Anderson [18] using a cold neutron backscattering spectrometer below the transition and a time of flight instrument above it. Figure 9 shows tħe quasielastic widths as a function of *Q* at 660 and 720 K. These energy widths were determined by fitting the data taken at a particular value of the scattering vector to a Lorenztian convoluted with the instrumental resolution. The solid line in Fig. 9a is a fit to a model which assumes that the diffusion occurs by instantaneous jumps between the nearest Li sites on the ordered sublattice as shown by the vector *ℓ*_2_ in the insert. For the much more rapid Li diffusion in the lattice gas phase, the data can be fit assuming that the jumps occur between the nearest neighbor commensurate sites shown by vector *ℓ*_1_ in the insert. In addition to the diffusional mechanism these fits yield values of the diffusion constant of l× 10^−4^ mm^2^/s and 24×l0^−4^ mm^2^/s at 660 and 720 K, respectively. From the temperature dependence of the diffusion constant in the ordered phase one obtains an activation energy of (1.0±0.3)eV. These results demonstrate the ability to “tune in” different diffusional processes with different neutron scattering spectrometers which operate in different dynamical ranges.

##### Self-Diffusion in bcc β-Titanium

**Fig. 10:**
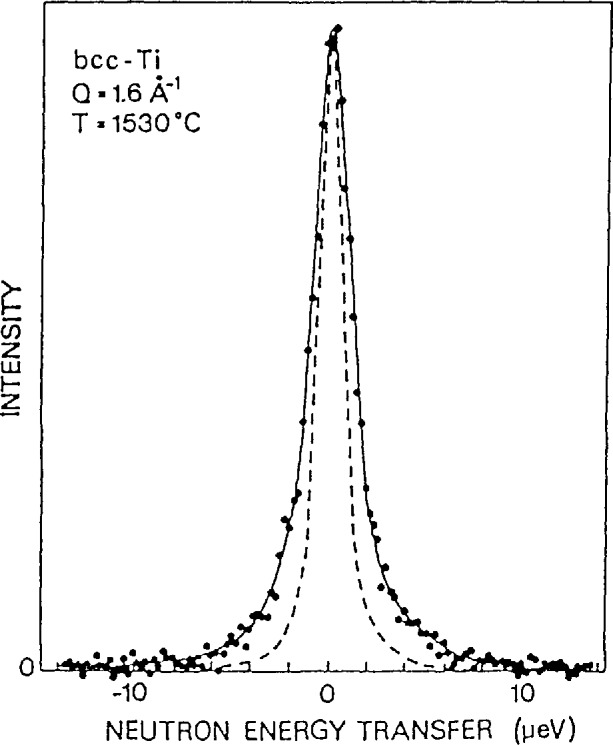
Quasielastic spectra from bcc Ti at *Q* = 1.6 Å^−1^ and *T* = 1530 °C. The solid line is a fit to a single Lorentzian convoluted with the instrumental resolution. The dashed line represents the measured resolution [19,20].

**Fig. 11:**
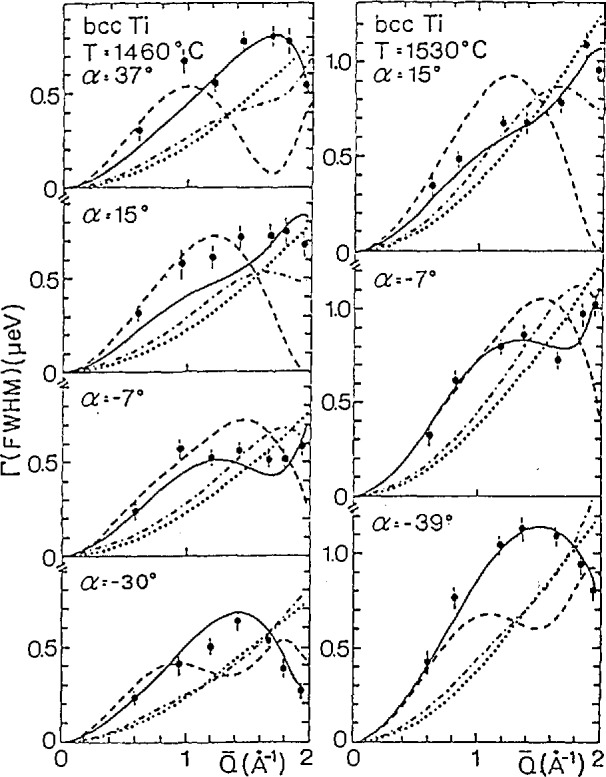
Linewidths at the quasielastic neutron spectra of bcc **Ti** at 1460 °C (left) and 1530 °C (right) as a function of *Q*. Left: scattering plane parallel to the (001) crystal plane. Right: scattering plane parallel to the (012) crystal plane, *α* denotes a rotation of the sample around an axis perpendicular to the scattering plane and is defined as the angle between the incident neutrons and the <100> crystal direction. The model calculations are: solid line, l/2[111] nearest neighbor jumps; dashed line, [100] 2nd nearest neighbor jumps; dotted line, tetrahedral interstitial jumps [19,20].

When plotted as a function of *T*_m_/*T*, (where *T*_m_ is the melting temperature) self diffusion in the group IVb metals (*T*i, Zr, and Hf) is orders of magnitude faster than for other bcc metals. In order to determine the diffusional mechanism Vogl et al. [19,20] have performed an exquisite measurement of the quasielastic scattering due to self diffusion in a single crystal of bcc Ti. A typical spectrum, along with a fit assuming a single Lorentzian convoluted with the experimental resolution is shown in Fig. 10. Figure 11 shows the *Q* dependence of the widths for two temperatures and several different crystal orientations. The solid lines represent fits to an encounter model of 1/2[111]NN jumps. In a model of this type only the jump vector between the original and final sites is relevant. The details of what happens in between are forgotten. The dashed lines in Fig. 11 represent an encounter model description of [100]NN jumps, while the dotted and dashed lines represent standard descriptions of tetrahedral and octahedral interstitial jumps respectively. Clearly this data reveals that the self diffusion of Ti in *β*-Ti is dominated by 1/2[111] jumps into nearest neighbor vacancies, however a small additional fraction of jumps into second nearest neighbor positions is also consistent with the data.

##### Reorientations of Benzene

**Fig. 12:**
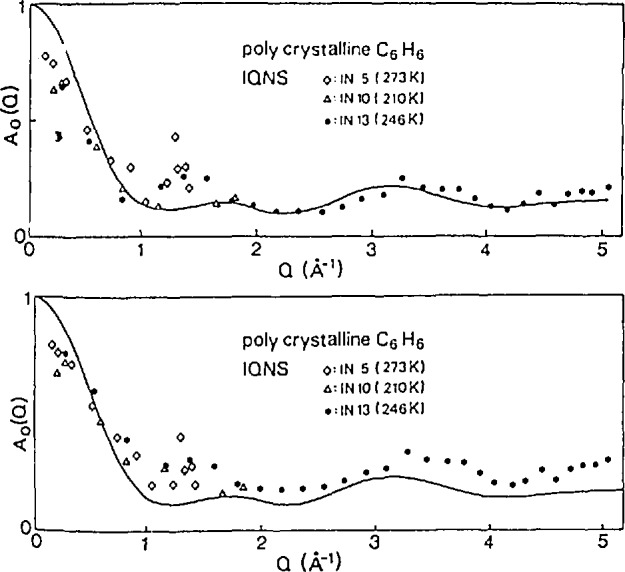
The elastic incoherent structure factor obtained by a model in which the benzene molecule undergoes rotational jumps of 60° (top) and by a model in which 60°, 120°, and 180° jumps are equally probable (bottom). The neutron scattering data clearly demonstrates that 60° jumps are the predominant rotational mechanism. The disagreement between the data and the model at small *Q*’s is due to multiple scattering effcets [21].

The previous two examples described systems in which the diffusing atoms (Li or Ti) undergo long-range translational motion so that the scattering law consists of a single Lorentzian component. As shown earlier, if the quasielastic scattering is due to rotational jumps, the scattering law is the sum of a δ-function and one or more Lorentzians. An interesting example of this type of system is crystalline benzene which has recently been studied using cold neutron backscatter-ing methods by Fujara et al. [21]. The EISF determined at 210 K by fitting the data to a model of random 60° jumps is shown in Fig. 12. The solid line is a calculation of the EISF assuming sixfold rotational jumps and that the radius of the ring of H atoms is 2.479 Å. The small disagreement at low *Q* can be attributed to multiple scattering effects. Attempts were also made to fit the data to a model which allowed for 120° and 180° jumps in addition to 60° jumps with equal probabilities. However, the EISF determined using this model was consistently larger than expected for sixfold rotations. Thus, these data show that benzene rotates principally by 60° jumps with a correlation time of approximately 30 ns at 210 K. In addition the temperature dependence of the correlation time was found to be consistent with an activation energy of 182 meV determined using NMR.

##### Dilute and Semidilute Polymer Solutions

**Fig. 13:**
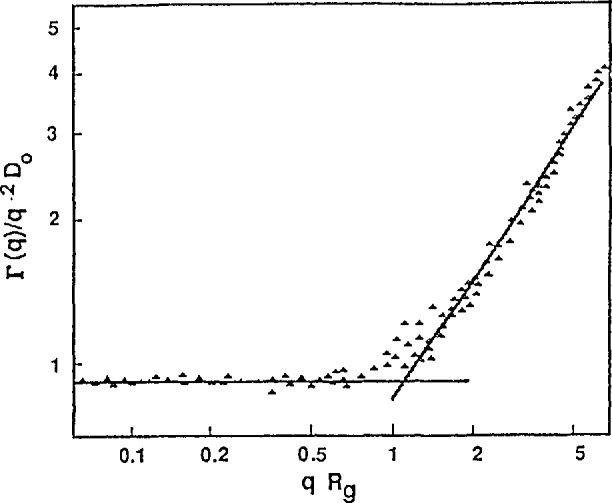
Quasielastic light scattering from polystyrene in various solvents showing the crossover from the *Q*^2^ to the *Q*^3^ scaling law (small to intermediate *Q* for the first cumulant) [16.]

**Fig. 14:**
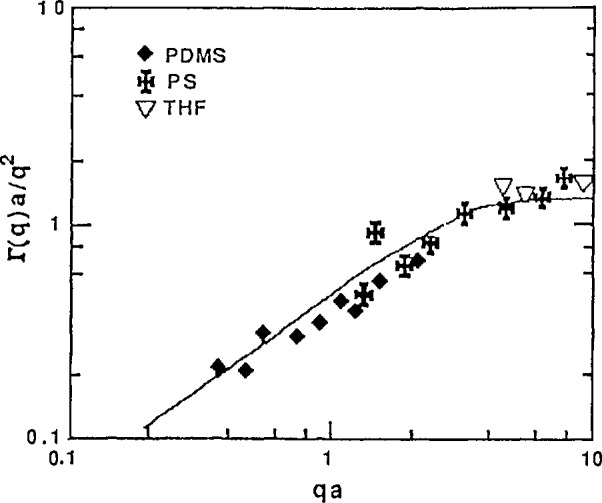
Neutron spin echo spectra from polystyrene in CS_2_ showing the crossover from the *Q*^3^ to the *Q*^2^ scaling law (intermediate to high *Q*) for the first cumulant.

In dilute (monodisperse) polymer solutions, and at the small *Q* limit (QELS), *Γ*(*Q*) shows a characteristic *Q*^2^ dependence seen in Eq. [9]:

$$\underset{Q\to 0}{\mathrm{Lim}}\Gamma \left(Q\right)=Q^{2}D,$$ (29)

which describes the overall diffusion of the whole polymer chain with a diffusion coefficient *D*. At intermediate values of *Q* (where scattering is probing length scales smaller than the chain but larger than the monomer size), a *Q*^3^ dependence of *Γ*(*Q*) characterizes the Zimm (internal) Brownian diffusive modes [15,22]:

$$\Gamma \left(Q\right)=C\left(k_{B}T/\eta_{0}\right)Q^{3},$$ (30)

where *k*_B_*T* is the solution temperature in energy units, η_υ_ is the solvent viscosity and *C* is a numerical constant that depends on solvent quality and preaveraging of the hydrodynamic interaction (for Theta solvents, C = l/6π or C = 1/16 depending on whether hydrodynamic interactions are preaver-aged or not). The *Q*^2^ to *Q*^3^ transition has been observed [16] for polystyrene in various solvents (see Fig. 13). Moreover, at high *Q*, diffusion of a single monomer dominates and the Q^2^ law is recovered again. This last transition (*Q*^3^ to *Q*^2^) involves scattering vectors that can be reached only with NSE [23,24] as shown in Fig. 14.

In semidilute solutions (where individual polymer chains start overlapping each other) hydrodynamic interactions between monomers start being screened so that a Rouse description [25] of polymer dynamics is more appropriate. Moreover, excluded volume effects remain important only between entanglement points so that monomers that are topologically farther apart do not feel each other even if they belong to the same chain. Scaling ideas based on concentration and temperature “blobs” [26] have been successful in describing both static and dynamic properties of polymer solutions. In the case of a three-component polymer system (two polymers and a solvent for example), two characteristic relaxation times are observed [27,28]: a slow mode representing cooperative diffusion and a fast mode representing inter-diffusion.

##### Concentrated Polymer Solutions and Melts

In contrast to the case of dilute solutions, concentrated polymer solutions and polymer melt dynamics are dominated by interchain correlations. At a length scale smaller than the average distance between two entanglement points, the Rouse model (which neglects hydrodynamic interactions) describes chain dynamics well. The *Q*^3^ power law dependence of *Γ*(*Q*) in the intermediate *Q* region becomes a *Q*^4^ dependence [29]. However, at longer scale lengths, entanglements constrain the chain motion to occur in a “tube” created by surrounding chains. This is the reptation idea [30,31] whereby polymer chains perform a snake like diffusive motion by which they renew their configurations. Reptation corresponds to the slow mode (that was mentioned for semidilute solutions) at high *Q*. In crosslinked gels, for example, this slow mode disappears so that only fast modes describing local monomer motions remain. Tests of the reptation idea have brought about a better understanding of the viscoelastic behavior of polymer systems.

A dynamic Random Phase Approximation (RPA) approach [32,33] has been used to understand the effect of monomer interactions on the various diffusive normal modes in polymer multi-component melts. Within this framework, the intermediate scattering function *I*(*Q,t*) and its initial slope *Γ*(*Q*) can be related to their bare (i.e., when no interactions are present) counterparts. This approach has permitted the sorting out of the various diffusion coefficients (self, mutual, inter-, cooperative, etc.) that are measured by various techniques in various experimental (concentration, molecular weight) conditions. Self and mutual diffusion coefficients are measured in dilute polymer solutions and correspond to the diffusion of a single chain and to that of many chains respectively. Inter-diffusion and cooperative diffusion characterize ternary polymer systems comprising, for example, concentrated solutions of two polymers A and B and correspond to the fast and slow modes when taken at the proper limits. The interdiffusion coefficient represents the diffusion of A relative to B while the cooperative diffusion coefficient describes the diffusion of the polymers (both of A and B) in the solvent. Interdiffusion for instance is the dominant mode in phase decomposing blends. It is interesting to note [28] that for the case of a diblock copolymer in solution, the first cumulant for the interdiffusion mode remains finite at the *Q*→0 limit (contrary to the definition of a diffusive mode). This is reminiscent of the “optical mode” in multilayer crystalline solids keeping in mind, of course, that the interdiffusion mode is nonpropa-gating.

Effects of temperature, concentration, molecular weight and chain stiffness on the dynamics of polymer solutions have been investigated. The molecular weight dependence of the mutual diffusion coefficient changes from *N*^−1/2^ with hydrodynamic interactions (dilute solutions) *io N*^−1^ in concentrated solutions of short chains in long chains (Rouse dynamics) to *N*^−2^ for melts of long chains (where reptation dominates). Recall that *N* is the number of monomers per polymer chain. The NSE technique is also particularly useful in observing polymer chain stiffness [23,34] and its effects on diffusion at intermediate and high *Q* values.

##### Equilibrating Polymer Blends

The previous sections described the dynamics of polymer systems that are in thermal equilibrium. The observed (Zimm, Rouse Reptation) modes are due to Brow-nian diffusion in solutions and in melts. This section, however, briefly describes “real time” dynamics of polymer systems following gradients in temperature [35] (such as across phase transitions) or in concentrations [36] (two films are superposed face-to-face and allowed to diffuse into each other upon heating). In the first case, the crossing of the phase boundary could be towards equilibration (from two-phase into the miscible region) or towards growth (the other way around). The time scales involved are ideally suited for investigation by quasielastic scattering methods.

The intermediate scattering function *I*(*Q,t*) described in the previous sections involves time correlations of the fluctuating density *ρ*(*Q,t*) (in Fourier space):

$$I\left(Q,t\right)=\left(1/N_{P}N^{2}\right)<\rho \left(-Q,0\right)\rho \left(Q,t\right)>.$$ (31)

For equilibration/growth processes, what is measured instead is the time evolution of *ρ*(*Q,t*):

$$S_{t}\left(Q\right)=\left(1/N_{P}N^{2}\right)<{\left|\rho \left(Q,t\right)\right|}^{2}>.$$ (32)

The Cahn-Hilliard-Cook theory [37,38] describes small deviations from equilibrium (early stage of spinodal decomposition) and can predict decay rates *R*(*Q*) of the time dependent structure factor:

$$\begin{array}{l} S_{t}\left(Q\right)=\left[S_{0}\left(Q\right)-S_{\infty }\left(Q\right)\right]\exp\left[-2R\left(Q\right)t\right]+\\ S_{\infty }\left(Q\right). \end{array}$$ (33)

Here, *S_υ_*(*Q*) and *S_∞_*(*Q*) are the initial and final (Virtual) values of the structure factor *S_t_*(*Q*). When the concentration fluctuations are small, *R*(*Q*) can be simply expressed in terms of the mobility *M*, the interfacial free energy coefficient *K* and the inter-diffusion coefficient *D*_int_:

$$R\left(Q\right)=Q^{2}D_{\mathrm{int}}-2Q^{4}MK.$$ (34)

These temperature jump experiments [35] are actually a means to measure *D*_int_. Two main theories describe the molecular weight dependence of *D*_int_: a “slow mode” theory [39,40] based on the incom-pressibility assumption and a “fast mode” theory [41] assuming vacancies present in the relaxing blend. The fast mode theory predicts an additive superposition of the mobilities of each component while the slow mode theory predicts an additivity law for the inverse of the mobilities. This is a new area of research (with ongoing controversies) where quasielastic scattering methods are the main research tools.

### 2.3 Tunneling Spectroscopy

#### 2.3.1 Basic Theory

**Fig. 15:**
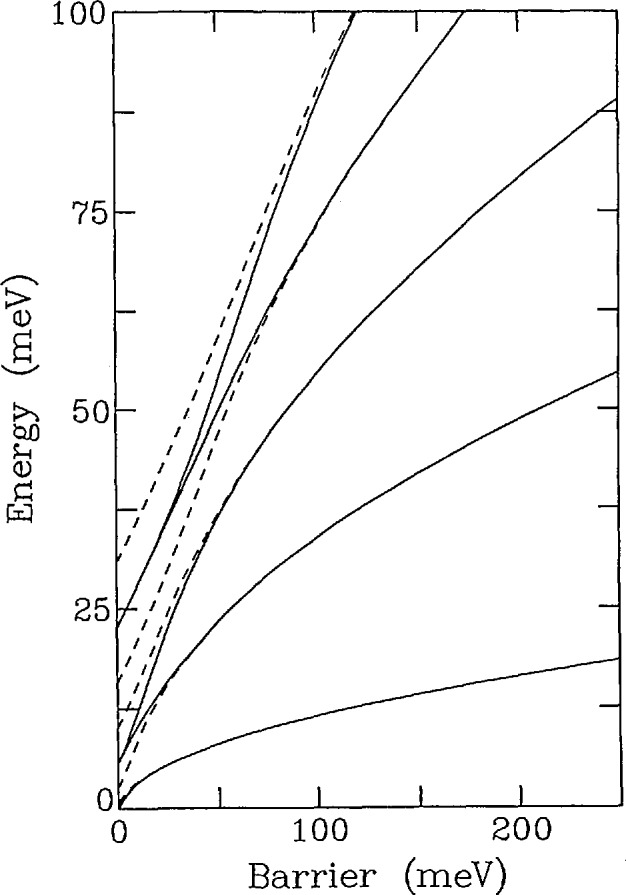
Energy levels as a function of *θ* the barrier for methyl groups in a threefold cosine potential. The dashed lines represent singly degenerate states (A symmetry) while the solid lines are doubly degenerate states (E symmetry).

**Fig. 16:**
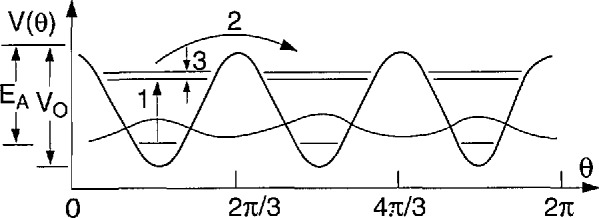
Schematic diagram of the energy levels in a threefold cosine potential. *V*_0_ is the barrier and *E*_A_ is the classical activation energy. Also shown are schematic wavefunctions in the ground state and transitions from the first excited state.

**Fig. 17:**
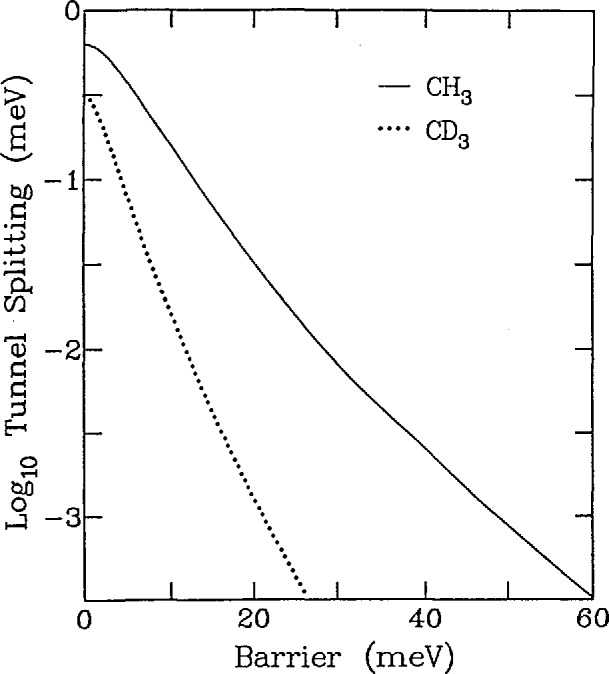
Semilogarithmic plot of the ground state tunnel splitting of a methyl group in a threefold cosine potential versus the barrier to rotation. The solid line is for a hydrogenated methyl group while the dotted line is for a deutcratcd one.

Perhaps the phenomena most studied using cold neutron backscattering is the rotational tunneling of small molecules and polyatomic ions in solids. In order to understand the origin of this effect consider a molecule in an m-fold potential *V_m_*(*θ*) given by

$$V_{m}\left(\theta \right)=\frac{V_{0}}{2}\left[1-\cos\left(m\;\theta \right)\right],$$ (35)

where *V*_0_ is the height of the barrier and *θ* is the rotational angle. The Schrödinger equation is then given by

$$\left[-B\frac{\partial^{2}}{\partial \theta^{2}}+V_{m}\left(\theta \right)\right]\psi_{n}=E_{n}\psi_{n}$$ (36)

where *B =ħ*/2*I* (*I* is the moment of inertia), *ψ_n_* is the wavefunction, and *E_n_* are the energy levels. Figure 15 shows the energy levels which are solutions to this equation as a function of the barrier height *V*_0_ assuming a threefold potential and that the rotating species is a methyl (CH_3_) group. Here the solid lines represent singly degenerate levels having A symmetry while the dashed lines correspond to doubly degenerate solutions of *E* symmetry. Basically there are three regimes. The first is where the barrier is zero which corresponds to free quantum rotors characterized by doubly degenerate levels having energies *E_j_* ∝ *j*^2^ where *j* is the rotational quantum number. The second is the limit of large barriers where the molecule or ion undergoes harmonic librations which are characterized by triply degenerate levels with *E_n_* ∝ (*n* + 1/2). Here *n* is the librational quantum number. Perhaps the most interesting region is that between these two extremes which is characterized by the tunnel splitting of the librational ground state and of the excited states resulting from the overlap of the wavefunctions shown schematically in Fig. 16. This splitting is quite sensitive to *V*_0_ since the overlap of the wave-functions depends exponentially on the barrier. This approximate exponential dependence of the ground state tunnel splitting is shown for both CH_3_ and CD_3_ in Fig. 17. Thus, tunneling spectroscopy is capable of yielding extremely detailed information on interatomic potentials in solids.

#### 2.3.2 Applications

##### Nitromethane

One of the most interesting applications of tunneling spectroscopy is the determination of the rotational potential felt by the methyl group in solid nitromethane (CH_3_NO_2_). Nitromethane is an ideal candidate for such studies for several reasons. First the internal barrier to rotation is very small. Thus in the solid phase, intermolecular interactions will dominate the rotational potential. In addition, the molecule is a simple one and will display only one-dimensional rotation. Finally, diffraction studies have shown that the space group of the crystalline material is P2_1_2_1_2_1_ which has only one molecule in the asymmetric unit [42]. Therefore, all methyl groups have the same environment and there is only one rotational potential to be determined. Diffraction studies have also demonstrated that no phase transitions occur between 4.2 K and the melting point of 244.7 K [42].

**Fig. 18:**
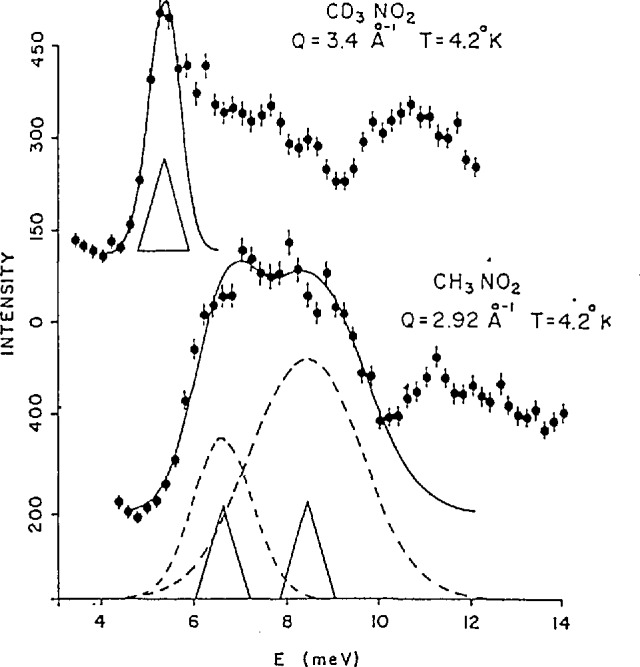
Inelastic neutron scattering spectra showing the transition from the ground state to the first excited state in CH_3_NO_2_ (6.7 meV) and CD_3_NO_2_ (5.3 meV) [43].

**Fig. 19:**
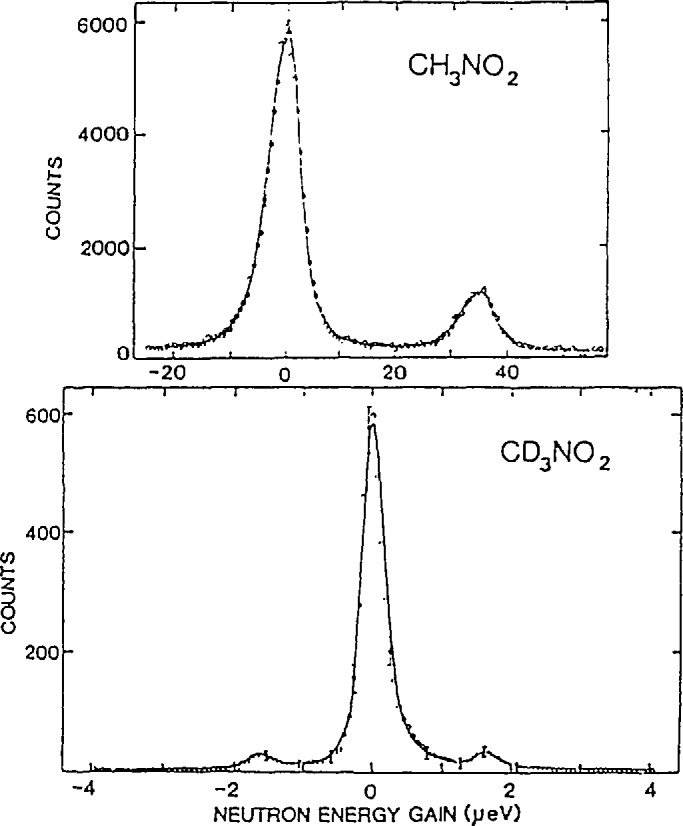
Measurements of the tunnel splitting for CH_3_NO_2_ and CD_3_NQ_2_. [45].

**Fig. 20:**
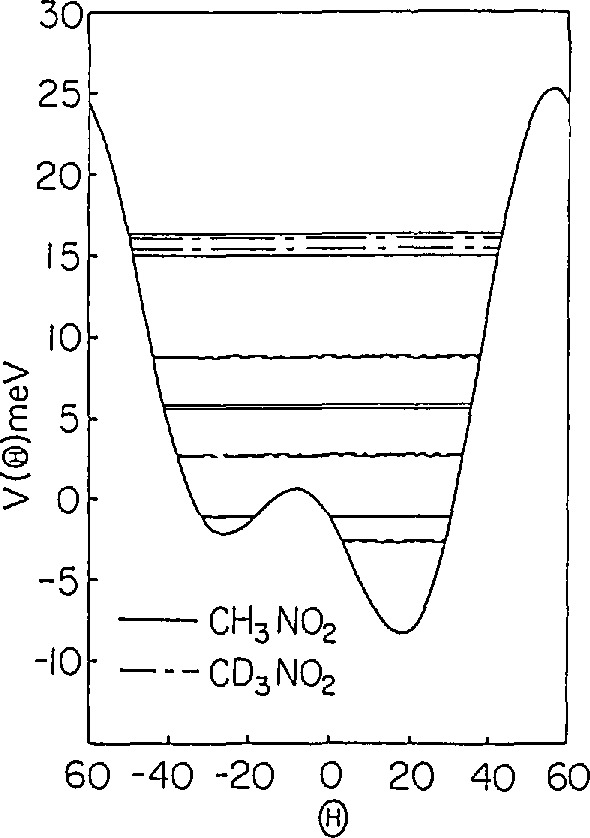
Potential determined by Cavagnat et al. [46] which is consistent with all of the spectroscopic data. The solid lines are the energy levels for CH_3_NO_2_.

The inelastic neutron scattering measurements of the transitions between the ground state and the first excited state obtained by Trevino [43] at 4.2 K are shown for both CH_3_NO_2_ and CD_3_NO_2_ in Fig. 18. From these spectra and from the temperature dependence of these spectra, the transition to the first excited state could be assigned to the peaks at 6.7 and 5.3 meV for the hydrogenated and the deuterated compounds respectively. Note that these values do not simply vary as $1/\sqrt{m}$ which indicates that the potential is quite anharmonic. Further measurements also revealed a transition to the second excited state at 17.5 meV for CH_3_NO_2_ and 10.6 for CD_3_NO_2_. The most important results for the characterization of the potential are the measurements of the ground state tunnel splittings for both samples performed by Trevino and co-workers [44,45] which are shown in Fig. 19. Here one observes clear transitions at 35 and 1.7 µeV for the hydrogenated and deuterated systems, respectively. Taken together these spectroscopic results are inconsistent with a simple threefold potential. However, it was shown by Cavagnat et al. [46] that a potential of the form

$$V\left(\theta \right)=V_{3}\left[1-\cos\left(3\theta \right)\right]+V_{6}\left[1-\cos\left(6\theta +\delta \right)\right]$$ (37)

would completely describe all of the spectroscopic results when *V*_3_ = 25.5 meV, *V*_6_= − 15.5 meV and δ = 30°. This potential is shown in Fig. 20 and the calculated energy levels are given in Table 1. In addition, these authors showed that a potential of this form could be obtained by using a simple Lennard-Jones model to describe the interactions between the methyl hydrogens and the surrounding lattice fixed. These calculations indicated that the origin of the sixfold term in the potential is the repulsive H-O interaction and the asymmetric location of the sixfold term with respect to the threefold term is due to the asymmetric distribution of the oxygen atoms with respect to the equilibrium distribution of the methyl groups.

**Fig. 21:**
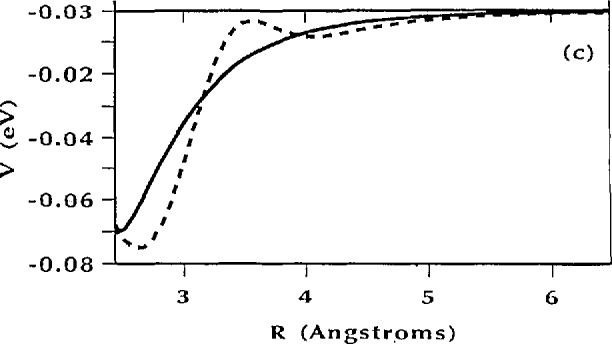
Lennard-Joncs H-O potential used by Cavagnat et al. [47] compared to the Gaussian-corrected Lennard-Jones potential obtained by Rice and Trevino which reproduces both the equilibrium orientation of the methyl group and the spectroscopy results.

Recently however, Rice and Trevino [47] have pointed out that the potential produced by this H-O interaction does not reproduce the equilibrium orientation of the methyl group. Guided by the maximum entropy method they were able to show that by including a small additional wiggle on the Lennard-Jones (Fig. 21) potential all aspects of both the spectroscopy and the structure, could be reproduced. Thus, through the use of a combination of thermal neutron spectroscopy and diffraction and cold-neutron spectroscopy a detailed description of the H-O interaction in nitromethane has been determined.

##### Methyl Iodide

**Fig. 22:**
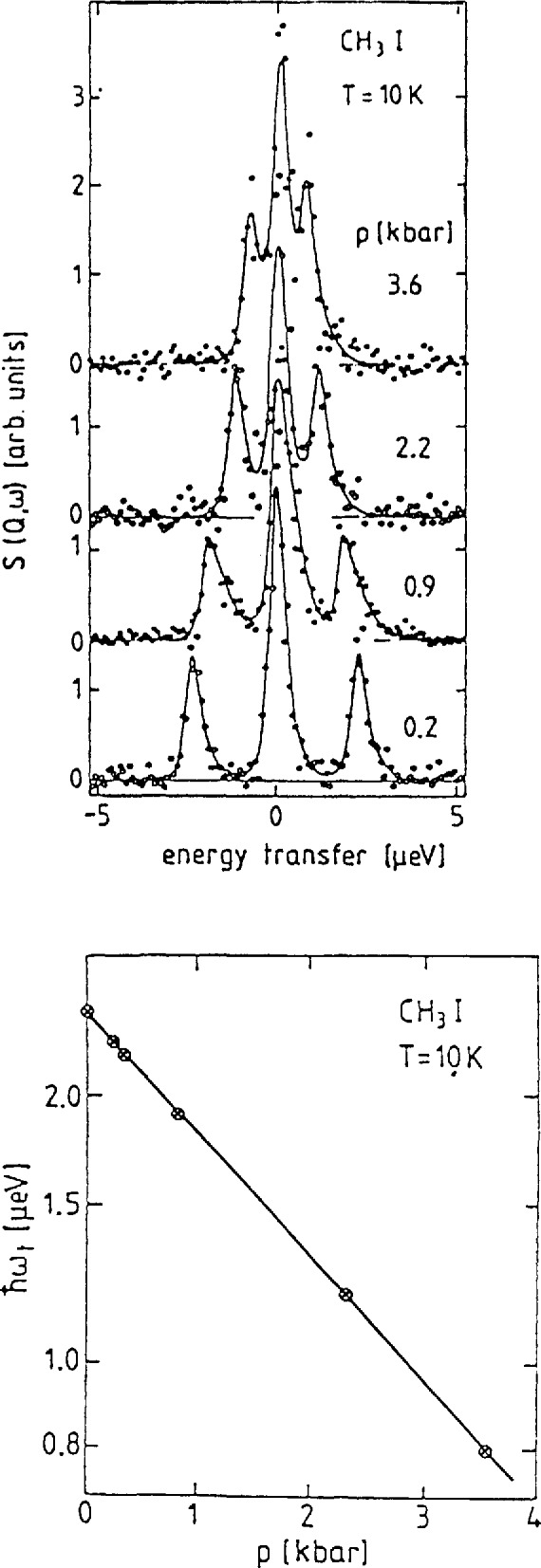
(a) Tunneling spectra in CH_3_I as a function of pressure, (b) Semilogarithmic plot of the tunneling energy as a function of pressure [49].

**Fig. 23:**
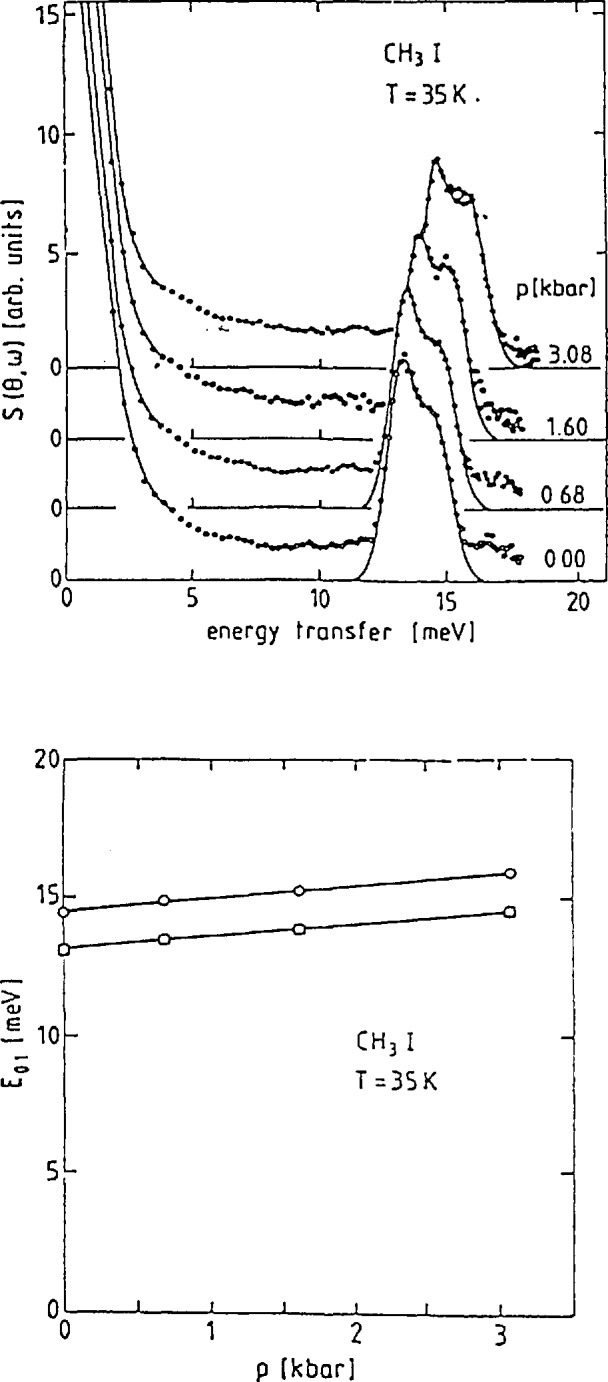
(a) Inelastic neutron scattering spectra of the transition from the ground state to the first excited state as a function of pressure, (b) Energy of the transition from the ground state to the first torsional level as function of pressure [49].

The pressure dependence of the tunneling transitions in CH_3_I is an excellent demonstration of the extreme sensitivity of these excitations to the rotational potential. Like nitromethane, methyl iodide has only one solid phase with only one molecule in the asymmetric unit cell. Therefore, all methyl groups feel the same potential and only one tunneling line will be observed. The spectra obtained using the backscattering technique are shown for several different pressures in Fig. 22a [48,49]. Note that the peaks decrease in energy as the pressure increases indicating an increase in the potential barrier. Fig. 22b shows that the ground state tunnel splitting depends exponentially on the pressure. This is to be contrasted with the results obtained for the energy of the transition to the first excited state shown in Fig. 23. The pressure dependence of this transition is linear and rather weak at that. Prager and coworkers [48,49] also showed that these spectra could be described by a potential of the form

$$V\left(\theta \right)=\frac{1}{2}V_{3}\;\left(1+\cos\;3\theta \right)+\frac{1}{2}V_{6}\left(1+\cos\;6\theta \right),$$ (38)

and that the threefold term accounts for ~92 percent of the total barrier, *V*_B_ *= V*_3_ *+ V*_6_. It is worth noting that the value of *V*_B_ determined from these measurements increases by < 20 percent from ambient pressure to 3 kbar while the tunnel splitting decreases by more than a factor of 2.5 clearly demonstrating the extreme sensitivity of this technique to details of the rotational potential.

## 3. CNRF Instruments

### 3.1 NIST Backscattering Spectrometer

**Fig. 24:**
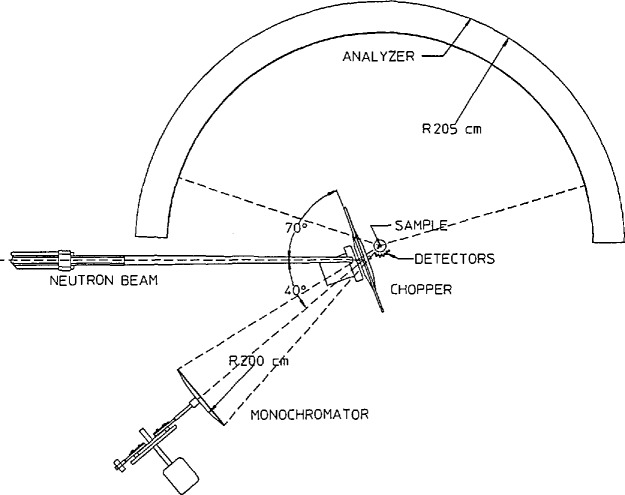
Schematic diagram of the CNRF backscattering spectrometer.

The cold neutron backscattering spectrometer (CNBS) which will be located in the CNRF is shown schematically in Fig. 24, (also see Table 1). The guide supplying the neutrons will have a cross sectional area of 6 × 15 cm^2^ and will have supermirror coatings on the tops and bottoms while the sides will be coated with ^58^Ni. The principal design goal has been to maximize the intensity of the instrument while maintaining an energy resolution of < 1 µeV. Thus the CNBS will have an energy resolution more than 10 times better than that of any instrument which currently exists in the United States. The first element of the instrument will be a wavelength selection device, the purpose of which is to suppress the background. This will consist of a Be filter, a Bi filter, possibly a velocity selector similar to that used on the small angle scattering and possibly a chopper with a duty cycle of about 1/2 to pulse the beam so that there are no neutrons striking the phase space chopper (which will be described in detail below) when neutrons are being counted in the detector.

The remaining neutrons will then pass through a converging supermirror guide which will compress the beam cross section from 6 ×15 cm^2^ to 3 × 3 cm^2^. It is not presently possible to quantify the length or the angle of convergence of the guide because of uncertainties in supermirror development; however it is hoped that an increase in flux of at least a factor of three will be possible. The neutrons will then impinge on a phase space transformer [50]. This can result in a substantial increase in neutron flux at the sample position because there is typically a substantial mismatch of the angular resolution of the primary and secondary sides of cold neutron backscattering instruments. This occurs because the divergence of the incident beam is limited by the neutron guide on which the instrument is installed, while the angular resolution of the secondary spectrometer is quite low due to the large area analyzing crystals and the detector geometry. Therefore it is possible to increase the flux at the sample position without degrading the energy resolution by increasing the angular divergence of the incident beam. This will be done to some extent through the use of the converging supermirror guide. However, it seems impossible to match the *Q* resolution of the primary spectrometer to that of the secondary spectrometer with current supermirror technology. To overcome this difficulty Schelten and Alefeld [50] have proposed a neutron phase space transformation which uses moving mosaic crystals to change a well-collimated, white neutron beam into a divergent, nearly monochromatic one. Physically, this occurs because the slower moving neutrons are diffracted at higher angles and therefore, get a “push” from the moving crystal, while the Bragg condition is satisfied at smaller angles for the faster neutrons causing diffraction to occur from crystallites moving away from the incident neutrons thereby reducing their speed.

We have performed Monte Carlo simulations of this device in order to determine the gain expected for parameters relevant to the CNBS at NIST. The beam which emerges from the converging supermirror guide will have a divergence η approximately twice the critical angle of Ni in the horizontal plane (*η_h_* = 2*θ_c_*). For the purposes of this simulation, the phase space crystal was chosen to be pyrolytic graphite (*d* = 3.354 Å and *θ*_0_=69.181°) with a thickness of 5 mm. The incident distribution of neutrons was taken to be a 65 K Maxwell-Boltzmann distribution (in accord with measurements of the flux from the cold source) truncated at 4 Å to simulate a Be filter in the incident beam and at 10 Å because wavelengths longer than this have essentially no probability of being diffracted by the moving crystal. This distribution was then multiplied by the square of the incident wavelength in order to account for the fact that *θ*_c_ is proportional to the wavelength. The horizontal and vertical mosaics and the velocity of the graphite crystals were included as input parameters. The reflectivity of graphite was accounted for with the Bacon-Lowde equation for diffraction from ideally imperfect crystals [51]. This will overestimate the reflectivity of the deflector crystal resulting in the simulated gains being somewhat larger than what one would actually observe. (Note that the reflectivity is a function of the crystal speed. This has been included.) All of these simulations have been performed using the assumption that Si (111) crystals will be used as the monochromator (λ ≈6.27 Å).

**Fig. 25:**
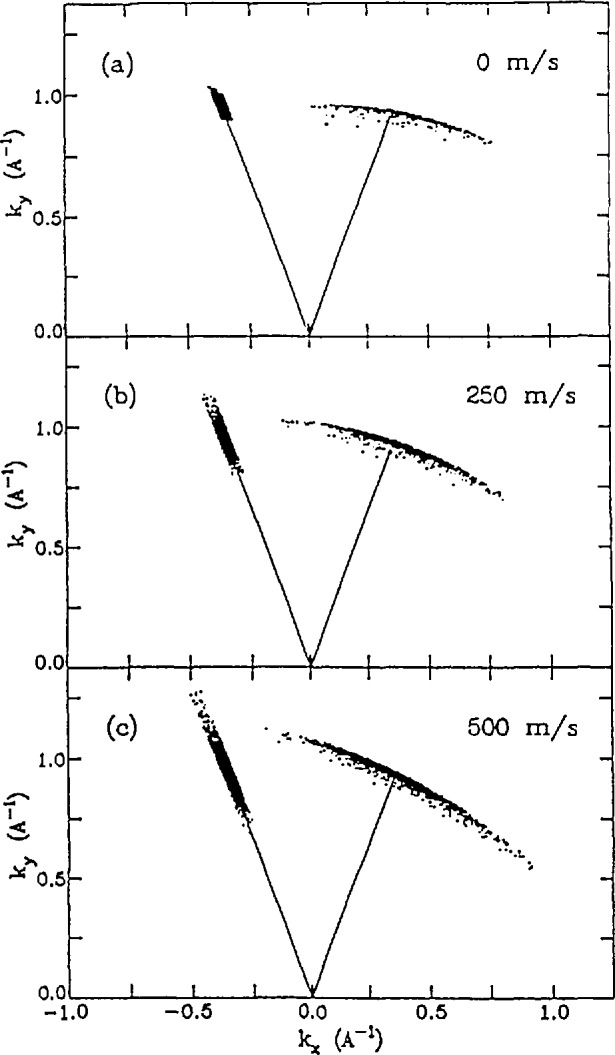
(a) Projection of a 3-d simulation of Bragg diffraetion from a stationary erystal with a mosaic of 10°. The dots represent the initial and final wave veetors of the diffraeted neutrons. (b) Same view for a erystal speed of 250 m/s. Note that the initial phase spaee volume has expanded and the final volume has been “tilted” compared to the results obtained for the stationary erystal. (c) Crystal speed = 500 m/s.

Two-dimensional projections of simulated Bragg reflections from a crystal having an isotropic 10° mosaic are shown in Fig. 25 for three different crystal speeds. Here the incident and final *k_x_* and *k_y_* values of the diffracted neutrons are represented by individual dots and the reference values are indicated by the solid lines. Two effects are evident. The first is that the phase space volume increases as the crystal velocity increases. This is because the Bragg reflection takes place at a lower angle in the Doppler frame. The second effect is that the diffracted beam tilts in phase space as the crystal velocity is changed. This tilt can be optimized so that the maximum number of neutrons have the correct energy to be backscattered from a Si (111) crystal. Note that this does not violate Liouville’s theorem because the orientation, not the volume, of the final phase space element has been changed.

**Fig. 26:**
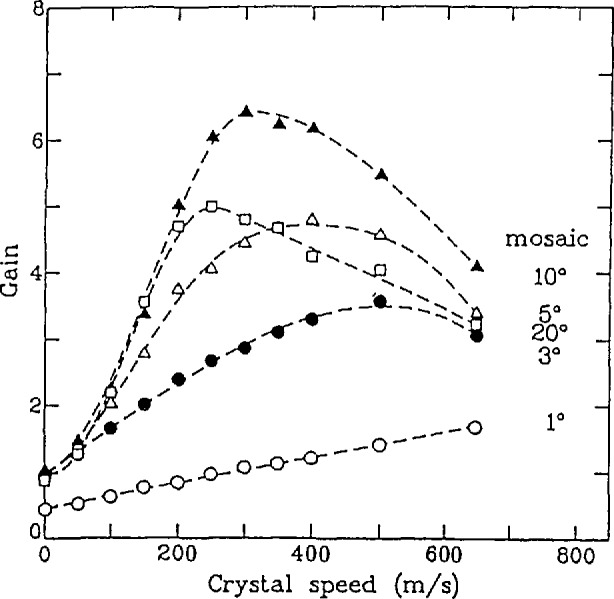
Peak intensity relative to that obtained for a stationary crystal. For a mosaic of 10°, the phase space transform leads to a gain of 6 for a crystal speed of 300 m/s. The dashed lines are guides to the eye.

The most important information from the standpoint of increasing the flux of backscattering instruments is displayed in Fig. 26. Here the peak intensity (relative to that obtained for a crystal velocity of zero) is shown as a function of speed for mosaics of 1°, 3°, 5°, 10°, and 20°. For mosaics of 3° or larger, the relative intensity increases from about 1 to a broad maximum, before decreasing again. For the parameters chosen here, the maximum gain is about 6 which occurs for crystals with a 10° mosaic moving at about 300 m/s. The results for a 20° crystal show a smaller gain due to the decrease in the reflectivity. The results for a crystal having a mosaic of 1° appear somewhat unique. Here the intensity increases linearly with the crystal speed and has a relative value of only about 1/2 for a speed of 0. This is because a 1° mosaic is too small when compared to the divergence of the incident beam.

After deflecting from the phase space transformer, the neutrons will travel approximately 2 m to the focussing monochromator crystals which will be mounted on a Doppler drive. The Doppler motion is necessary to vary the energy of the neutrons which impinge on the sample. The total range of energies available is determined by the maximum velocity of the Doppler drive. The NIST instrument will have a variable energy range (up to a maximum of ~100 μeV). In order to obtain the maximum flux, the neutrons will be focussed by the monochromator onto the sample. However, Fig. 24 shows that the sample is slightly displaced from the phase space transformer. Thus this results in a small deviation of the backscattering condition. To match this small worsening of the resolution, the monochromator will not be perfect Si, but possibly boron-doped Si. This will result in an increased value of *Δd/d* (see Sec. 2) and therefore increased intensity. A similar result could be achieved by bowing the individual Si crystals, however this results in a more Lorentzian resolution function while the doped crystals result in a more Gaussian lineshape [52]. Note that the level of boron in the sample would not result in significant loss of intensity due to absorption. A Ge_0.1_ Si_0.9_ monochromator which displaces the elastic peak by ~ 15 μeV will also be available.

After reflecting from the monochromator, the neutrons will pass back through the phase space transformer. This is possible because only half of the chopper is covered with graphite while the other half is transparent to neutrons. Furthermore, the frequency of the chopper is chosen so that time required for the neutrons to travel from the transformer to the monochromator and return is the same as that for the crystals to move out of the beam.

The neutrons are then scattered from the sample to the large (~9 m^2^) bank of boron-doped Si analyzer crystals, which focus the neutrons back to the detectors. The focussing and boron doping will be chosen so that the energy resolution of the secondary spectrometer matches that of the primary spectrometer. The detectors are electronically gated so that they are off when neutrons are striking the sample thereby avoiding direct scattering of neutrons from the sample to the detector. The energy transfer is ascertained by measuring the time of arrival of the neutrons at the detector. Since the total distance between the monochromator and sample and the energy of the scattered neutrons are known, the initial energy and therefore the energy transferred by the scattering from the sample can be determined.

Because the detectors are near the incident beam, the most important potential problem with this design is background. If this problem cannot be reduced to acceptable levels by reducing the spread in incident energies, thereby limiting the number of neutrons in the incident beam or by improving the shielding of the detectors, the detectors will have to be moved farther from the guide by placing a deflector in the guide. This will reduce the effectiveness of the phase space transformer so this will only be done as a last resort. However, contingency plans are in place for such an eventuality.

### 3.2 CNRF Spin Echo Spectrometer

The main components of the CNRF-NSE instrument which is still at a preliminary design stage (Fig. 2) are described here. Neutrons are first monochromated using a velocity selector and then polarized using a supermirror polarizer. Polarization in transmission geometry is preferred for colli-mation reasons (with reflection polarizers the whole instrument has to be able to rotate around the polarizer axis) although the transmission geometry is hampered by low polarization efficiencies. Next, the neutron spin direction is rotated from the horizontal forward axis to a vertical direction (using a *π*/2 flipper) more suitable for use in the first precession coil (solenoid) where the neutron magnetic moment precesses in a vertical plane. After the sample position, another precession coil makes the neutron spin precess in the other direction. In order to flip the spin direction, a *π* flipper is used between the two coils (just before the sample). Next, another *π/*2 flipper is required to rotate the magnetic moment from the radial direction to an axial one before reaching a spin analyzer (array of supermirrors). Finally, neutrons are detected in a position sensitive area detector.

In order for the NSE technique to work, the “field integral” (i.e., the integral of the magnetic field over the neutron path) must remain constant before and after the sample (therefore creating an “echo”). In practice, it is difficult to make exactly identical main coils to be used before and after the sample, so that besides the main coils, other smaller “correcting” coils are also used. Correction coils are added before the sample to optimize the echo and around the sample to correct for the earth magnetic field. No steel or magnetic materials can be used in making the coils or close to the instrument. Moreover, because very stable DC current supplies are required, current stabilities of the order of d*I*/*I* ~10^−5^ have to be achieved.

The CNRF-NSE spectrometer is in the preliminary design stage; detailed designs are planned to start soon.

## 4. Summary

We have presented the operating principles of two ultrahigh energy resolution neutron scattering spectrometers, the backscattering spectrometer and the spin echo spectrometer and have described types of measurements which can be done with these instruments at the Cold Neutron Research Facility at NIST. We have also discussed the basic design of the cold neutron backscattering spectrometer to be built in the CNRF. This information will assist researchers who are considering ultrahigh energy resolution neutron scattering experiments at NIST.

## Figures and Tables

**Table 1:** Characteristics of the backscattering spectrometer being designed for installation in the CNRF

Energy resolution	~0.75 μeV
Maximum energy range	~100 μeV
Neutron energy	2.08 mcV
*Q* -Range	0.1–1.8 Å^−1^
Flux on the sample	~ 10^5^ neutrons/cm^2^ s
Maximum sample size	3 × 3 cm^2^
Sample-Monochromator distance	~2 m
Sample-Analyzer distance	~2 m
